# Phospho-sulindac inhibits pancreatic cancer growth: NFATc1 as a drug resistance candidate

**DOI:** 10.3892/ijo.2013.2190

**Published:** 2013-11-27

**Authors:** ONIKA T. MURRAY, CHI C. WONG, KVETOSLAVA VRANKOVA, BASIL RIGAS

**Affiliations:** Division of Cancer Prevention, Department of Medicine, Stony Brook University, Stony Brook, NY 11794-8173, USA

**Keywords:** phospho-sulindac, pancreatic cancer, non-steroidal anti-inflammatory drugs, nuclear factor of activated T-cells, cytoplasmic 1

## Abstract

Phospho-sulindac (P-S), a promising anticancer agent, is efficacious in pre-clinical models of human cancer and is apparently safe. Here, we studied the effect of P-S on pancreatic cancer growth. We found that P-S strongly inhibits the growth of human pancreatic cancer cells *in vitro*, is efficacious in inhibiting the growth of pancreatic xenografts in nude mice, and has an excellent safety profile. Microarray analysis revealed that P-S induced the expression of nuclear factor of activated T-cells, isoform c1 (NFATc1) gene. NFATc1, a calcineurin-responsive transcription factor associated with aggressive pancreatic cancer. The role of increased NFATc1 expression on the growth inhibitory effect of P-S on cancer growth was evaluated by silencing or by overexpressing it both *in vitro* and *in vivo*. We found that when the expression of NFATc1 was abrogated by RNAi, pancreatic cancer cells were more responsive to treatment with P-S. Conversely, over-expressing the NFATc1 gene made the pancreatic cancer cells less responsive to treatment with P-S. NFATc1 likely mediates drug resistance to P-S and is an unfavorable prognostic factor that predicts poor tumor response. We also demonstrated that NFATc1-mediated resistance can be overcome by cyclosporin A (CsA), an NFAT inhibitor, and that the combination of P-S and CsA synergistically inhibited pancreatic cancer cell growth. In conclusion, our preclinical data establish P-S as an efficacious drug for pancreatic cancer in preclinical models, which merits further evaluation.

## Introduction

Pancreatic cancer is a devastating disease with an overall median survival time of 4–6 months, making it the fourth major cause of cancer-related deaths in the US (1). Contributing to the lethality of the disease is its ability to grow undetected until it reaches a metastatic state, where surgery, the only curative option, has little effect (2,3). Despite advances in chemotherapy, its impact on long-term survival has been minimal. Thus, there remains a compelling need to develop new and efficacious chemotherapeutic agents for pancreatic cancer.

Non-steroidal anti-inflammatory drugs (NSAIDs) have demonstrated antineoplastic properties in pancreatic cancer; however, their efficacy and safety as anticancer agents are limited (4). For example, sulindac, alone or in combination with other drugs, modestly inhibited the growth of pancreatic cancer in pre-clinical models (5–8). Chronic use of sulindac, however, is associated with significant gastrointestinal and renal toxicity. Prompted by these considerations, our group has synthesized phospho-sulindac (P-S). A key structural feature of P-S is the covalent modification of the -COOH moiety, a major culprit for its gastrointestinal toxicity, with a diethylphosphate group via a linker. Phospho-sulindac (P-S) has demonstrated significant efficacy against colon cancer, substantially exceeding that of sulindac (9). P-S is also much safer than sulindac (9,10), especially regarding its ability to spare the gastroduodenal mucosa (11). Hence, we hypothesized that P-S would be efficacious against pancreatic cancer.

Nuclear factor of activated T-cells (NFAT) are a family of nuclear transcription factors primarily involved in the regulation of T-cell activation and differentiation (12). Recent studies, on the other hand, revealed non-canonical functions of NFAT in several malignancies, most notably in pancreatic cancer, where it drives cancer progression via the promotion of cell proliferation, invasion and angiogenesis (13). Aberrant regulation of NFAT thus contributes to drug resistance to diverse therapeutic agents (14,15).

In the current study, we demonstrate that P-S inhibits pancreatic cancer growth *in vitro* and *in vivo*. We identified NFATc1-dependent signaling as a mechanism of drug resistance in pancreatic cancer; and showed that the activation of NFATc1 is an unfavorable prognostic factor that predicts poor tumor response to P-S, which can be overcome by pharmacological intervention.

## Materials and methods

### Reagents

Phospho-sulindac (P-S) was synthesized as reported (16). Sulindac was purchased from Sigma (St. Louis, MO, USA). Annexin V was purchased from Invitrogen (Grand Island, NY, USA). Propidium iodide (PI) and 5-bromo-2’-deoxyuridine (BrdU) were obtained from BD Bioscience (San Jose, CA, USA). All general solvents and reagents were of HPLC grade or the highest grade commercially available.

### Cell lines

Human pancreatic cancer cell lines BxPC-3, Mia PaCa-2, Panc-1 and HPAF II were obtained from American Type Tissue Collection (ATCC, Manassas, VA, USA). Cells were grown at 37°C in 5% CO_2_ in the specific medium suggested by ATCC and supplemented with 10% fetal calf serum (Mediatech, Herndon, VA, USA), penicillin 50 U/ml and streptomycin 50 *μ*g/ml (Life Technologies, Grand Island, NY, USA).

NFATc1 knockdown BxPC-3 and Mia PaCa-2 cell lines were generated using MISSION Lentiviral Packaging Mix (Sigma-Aldrich, St. Louis, MO, USA). Cells were infected with the lentivirus and then subjected to 3 *μ*g/ml of puromycin to generate stable cell lines. Decreased levels of NFATc1 were confirmed by western blotting.

The NFATc1 Mia PaCa-2 overexpression cell line was generated using NFATc1 human cDNA clone from Origene (Rockville, MD, USA). Cells were transfected using Lipofectamine 2000 (Invitrogen, Carlsbad, CA, USA) and selected with 1 mg/ml of G418.

### Cytokinetic analyses

Cell viability was measured by the 3-(4,5-dimethylthiazol-2-yl)-2,5-diphenyltetrazoliumbromide assay following the protocol of the manufacturer (Roche Diagnostics, Indianapolis, IN, USA). Apoptosis was assessed by Annexin V/propidium iodide (PI) staining (Life Technologies). Cell proliferation was determined by the bromodeoxyuridine (BrdU) incorporation method (BD Biosciences, San Jose, CA, USA).

### Gene expression microarray

Mia PaCa-2 cells were treated with vehicle or 1.5×IC_50_ P-S for 30 min or 2 h. Total RNA was isolated from cell lines using an RNA Extraction kit (Qiagen Inc., Valencia, CA, USA). Samples were submitted to Genome Explorations (Memphis, TN, USA) and analyzed on the Affymetrix Human Genome U133 gene chip (Cleveland, OH, USA).

### Immunofluorescence

Cells were seeded in an 8-well glass chamber slide (Lab-Tek, Rochester, NY, USA). The following day, cells were treated with vehicle or P-S for 24 h. Cells were fixed in 4% paraformaldehyde (15 min). Block and NFATc1 antibody (Abcam, Cambridge, MA, USA) incubations were performed in PBS with 2% BSA and 0.3% Triton. Cells were mounted with Vectashield mounting media with diamidinophenylindole (DAPI; Vector Laboratories Inc., Burlingame, CA, USA) and imaged using a Zeiss Axioplan inverted fluorescence microscope (Thornwood, NY, USA).

### Western blotting

After treatment, cells were lysed on ice with 1% Triton lysis buffer containing 2.5 mmol/l 4-nitrophenylphosphate, 1% SDS and 0.25% sodium deoxycholate for 30 min. The cell lysate (25 *μ*g) was fractionated by SDS-electrophoresis gel and transferred onto a PVDF membrane. This was followed by immunoblotting with NFATc1 (Abcam), pERK, pAKT (Cell Signaling, Danvers, MA, USA), COX-2 (Cayman Chemical, Ann Arbor, MI, USA) and c-Myc (Santa Cruz, Santa Cruz, CA, USA) antibodies. Secondary antibodies conjugated with horseradish peroxidase (HRP) (Santa Cruz Biotechnology) were applied to the membrane, followed by development on X-ray film.

### qRT-PCR

Total RNA was isolated from cell lines using TRIzol reagent as per the instructions of the manufacturer (Life Technologies). Ten micrograms of RNA was used in reverse-transcription using random primers and the M-MLV Reverse Transcriptase kit (Sigma). The following primer pairs were used for mouse NFATc1: forward 5′-CCAGTCATC GGCGGGAAGAAGA-3′; reverse 5′-TATACACCCCCAG ACCGCATCAGC-3′.

### Pancreatic cancer xenografts

All animal experiments were approved by the Institutional Animal Care and Use Committee at the Stony Brook University. Six-week-old female BALB/c nude mice (Charles River, Wilmington, MA, USA) were xenografted subcutaneously with Mia PaCa-2 cells (1.5×10^6^ in 100 *μ*l PBS) in the right and left flank. The animals were then treated for 4 weeks with vehicle or P-S (100 mg/kg/day, p.o.) (n=6/group).

To study the effect of NFAT expression on drug response, animals were separated into 6 groups (n=6/group) and treated by with vehicle (corn oil, p.o.) or P-S (100 mg/kg/day, p.o.) for one week prior to the inoculation of wild-type, NFAT Kd or NFAT overexpressing Mia PaCa-2 cells into the right and left flank. Treatment continued for 4 weeks. For all studies, tumor size was measured using a digital microcaliper. Upon study completion, animals were euthanized and tumors collected, measured and embedded in OCT.

### Immunohistochemistry

Mia PaCa-2 xenograft tissue was fixed in 10% phosphate-buffered formalin for 16 h, dehydrated and embedded into paraffin blocks as previously described (17). Slides were prepared and stained for Ki-67 (Santa Cruz Biotechnology), TUNEL (Roche Applied Science, Indianapolis, IN, USA), COX-2 (Cayman Chemicals), or NFATc1 (Abcam) positive cells. A pathologist blinded to sample identity scored the number of positive cancer cells and the number of all (positive and negative) cancer cells to calculate the percentage of positive cells.

### Statistical analysis

Results from at least 3 independent experiments and expressed as the mean ± SD were analyzed by the Student’s t-test. p<0.05 was considered significant.

## Results

### P-S inhibits the growth of pancreatic cancer cells in vitro and in human pancreatic xenografts

Table I summarizes the 24, 48 and 72 h IC_50_ values of P-S as well as those of sulindac in a panel of 4 human pancreatic cancer cell lines: BxPC-3, Mia PaCa-2, Panc-1 and HPAF-II. In all four cell lines, P-S showed an enhanced potency when compared to sulindac that was time-dependent, becoming higher the longer the incubation period. At 24 h, P-S showed an average potency enhancement of 22.5-fold over sulindac; at 48 h, 36.0-fold; and at 72 h, 60.8-fold.

**Figure 1.**
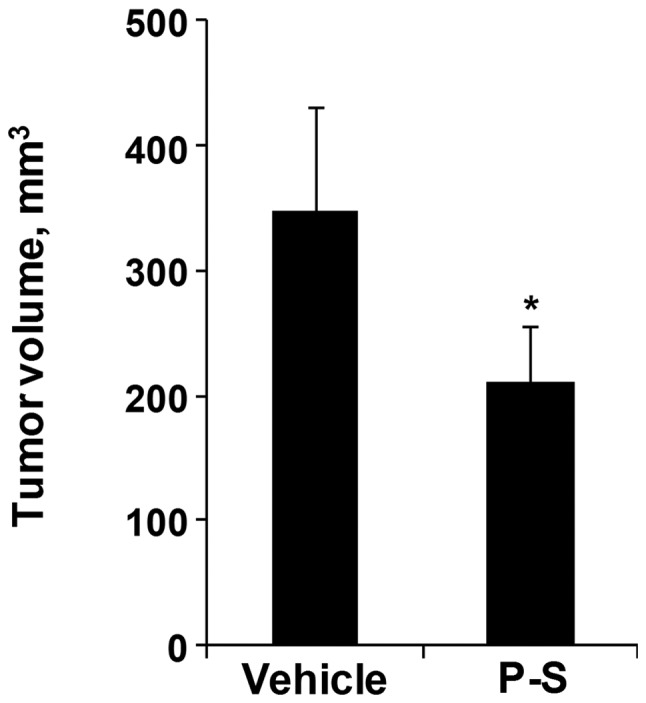
Efficacy of PS in Mia-PaCa-2 xenografts. Animals bearing Mia-PaCa-2 xenografts were treated by gavage with vehicle (corn oil) or P-S 100 mg/kg/day. Animals in vehicle and P-S treated groups had similar body weights. ^*^p<0.05, compared to control group.

We next evaluated the efficacy of P-S in nude mice bearing human pancreatic cancer cell xenografts. As shown in Fig. 1, tumor volume was decreased by 45.3% in P-S (p.o.) treated group in comparison to vehicle (p<0.05). Moreover, the P-S treated groups exhibit no changes in body weight or signs of toxicity. Thus, P-S is an efficacious drug in inhibiting pancreatic cancer growth *in vitro* and *in vivo*.

### The cytokinetic effect of P-S

**Figure 2.**
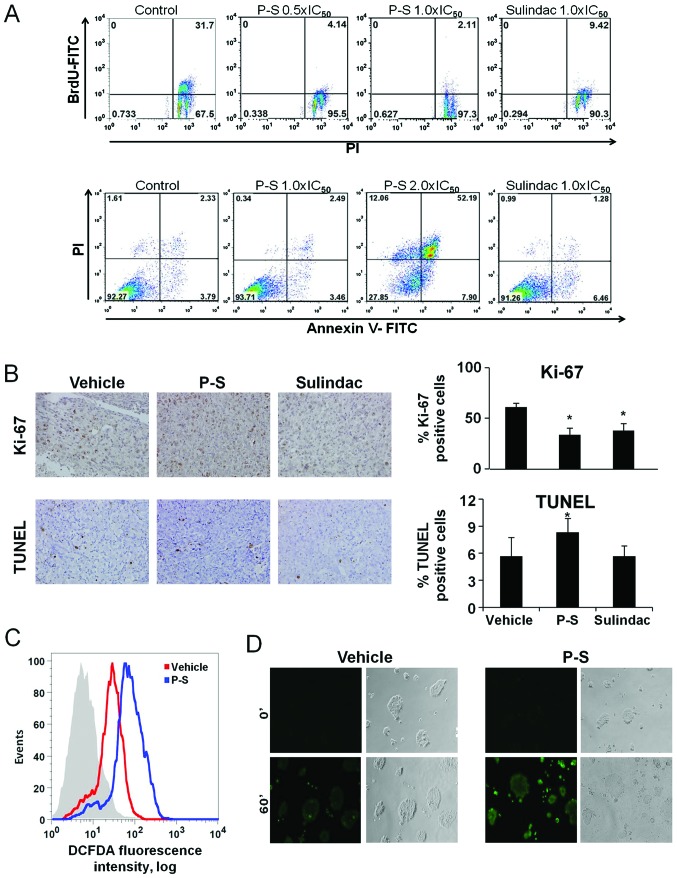
Effect of P-S on cytokinetics and ROS induction in pancreatic cancer cells. (A) BxPC-3 cells were treated with vehicle, P-S 0.5, 1.0 or 2.0×IC_50_ and sulindac 1.0×IC_50_ for 24 h. Cells were harvested, incubated with BrdU-FITC/PI (upper) or Annexin V-FITC/PI (lower) and analyzed by flow cytometry. P-S exerted a stronger cytokinetic effect than sulindac. (B) Mia PaC-2 pancreatic cancer xenograft tissue was analyzed by IHC using markers of cell proliferation (Ki-67) and apoptosis (TUNEL). (C) BxPC-3 cells were treated with vehicle or P-S 1.0×IC_50_ for 1 h, stained with 10 *μ*M DCFDA and analyzed by flow cytometry. (D) Live cell imaging for ROS levels in BxPC-3 cells. Cells seeded in 4-well chamber glass slides were treated with 10 *μ*M DCFDA and either vehicle or P-S for 1 h. There was a notable increase of ROS levels in cells treated with P-S. ^*^p<0.05, compared to control group.

Given that P-S suppresses the growth of pancreatic cancer cells, we determined the cytokinetic effect of P-S in BxPC-3 cells. P-S had a significant inhibitory effect on cell proliferation (Fig. 2A), reducing BrdUFITC(+) cells by 87.0% at 0.5×IC_50_, and 93.3% at 1.0×IC_50_. Sulindac 1.0×IC_50_ reduced BrdU(+) cells by 70.3%, 20% less in comparison to P-S 1.0×IC_50_. These results show that while both P-S and sulindac are able to reduce pancreatic cancer cell proliferation, P-S was more potent than its parent compound.

The effect of P-S on apoptosis was analyzed in BxPC-3 cells (Fig. 2A). There was no significant induction of apoptosis at P-S 1.0×IC_50_ or sulindac 1.0×IC_50_. However, P-S at 2.0×IC_50_ induced a dramatic increase in Annexin V-positive cells, with apoptotic cells comprising 60% of the total. These results show that P-S induces cell death in BxPC-3 cells in a concentration-dependent manner. Similar results were observed in Mia PaCa-2 cells (data not shown).

The cytokinetic effect of P-S on proliferation and apoptosis was analyzed *in vivo* (Fig. 2B). P-S inhibited cell proliferation, as indicated by the reduction in Ki-67 positive cells by 44.9% (p= 0.006) in Mia PaCa-2 xenografts. P-S also increased apoptosis in these xenografts. TUNEL positive cells were increased by 31.9% in comparison to vehicle.

### P-S induces ROS in BxPC-3 cells

Previous studies have shown that P-S induces reactive oxygen species (ROS) in various types of cancer cells (10,16). BxPC-3 cells were treated with P-S 1.0×IC_50_ for 1 h and ROS levels were determined using the DCFDA general ROS probe (Fig. 2C). There was a 3-fold increase in ROS in cells treated with P-S (p<0.05) compared to the vehicle. Live cell imaging for ROS levels was also performed in BxPC-3 cells treated either with vehicle or P-S for 1 h (Fig. 2D), using the DCFDA general ROS probe. Similarly, there was a notable increase in ROS in cells treated with P-S in comparison to vehicle.

### Microarray analysis of gene expression in Mia PaCa-2 cells treated with P-S

To further understand the signaling effects of Mia PaCa-2 cells, we analyzed gene expression of vehicle or P-S (1.5×IC_50_) treated cells using the Affymetrix Human Genome U133 gene chip. Gene expression of NFATc1 and AP1, members of the B- and T-cell receptor pathways, was upregulated >2-fold in response to treatment with P-S. NFATc1 is a member of the NFAT (nuclear factor of activated T-cells) family of transcription factors initially identified as regulators of T-lymphocyte activation (12,13). NFATc1 has been shown to be overexpressed in pancreatic cancer and contributing to the aggressive nature of the disease (12,13,20).

### Effect of P-S on the expression of NFATc1 and its downstream targets COX-2 and c-Myc in BxPC-3 and Mia-PaCa-2 cells

**Figure 3.**
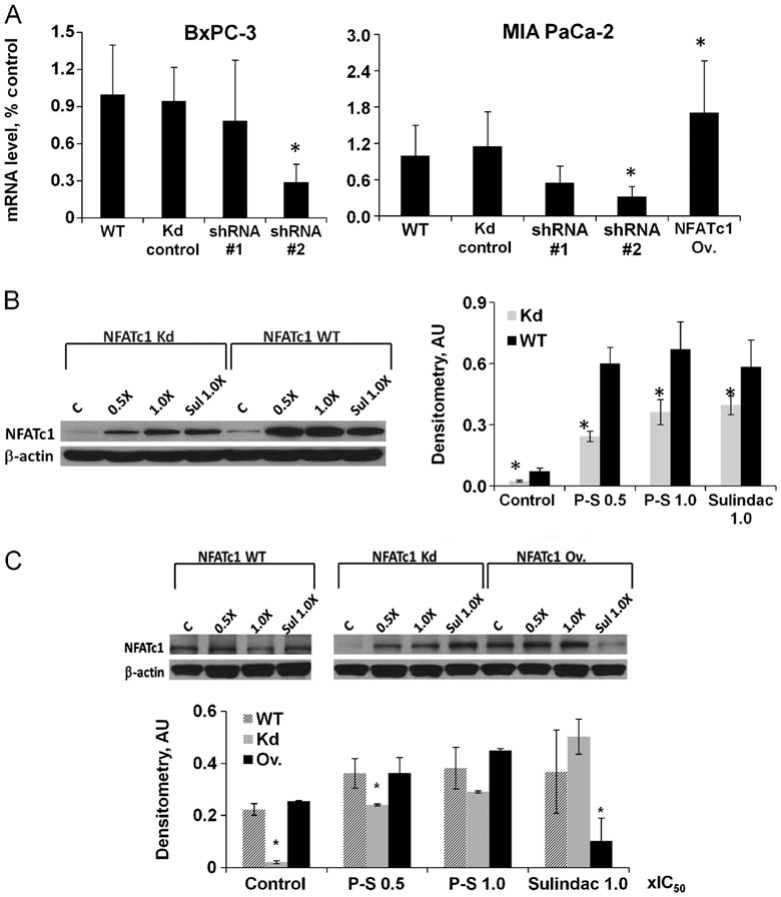
P-S induces the expression of NFATc1 in pancreatic cancer cells. (A) BxPC-3 and Mia PaCa-2 cells were transfected with NFATc1 shRNA or cDNA, clones selected and relative mRNA levels analyzed. (B) BxPC-3 NFATc1 WT and knockdown (Kd) cells were treated with vehicle, P-S 0.5 and 1.0×IC_50_, or sulindac 1.0×IC_50_ for 24 h. Cells were lysed and NFATc1 expression is analyzed by western blotting. (C) Mia PaCa-2 NFATc1 WT, Kd and overexpressing (Ov.) cells were treated with vehicle, P-S 0.5 and 1.0×IC_50_, or sulindac 1.0×IC_50_ for 24 h. Cells were lysed and NFATc1 expression is analyzed by western blotting. ^*^p<0.05, compared to control group.

Sustained activation of calcineurin-NFAT transcription pathway has a pro-proliferative effect in pancreatic cancer cells through the transcription activation of oncogenic c-myc (20) and cyclooxygenase-2 (COX-2) (21). To investigate the effect of P-S on NFATc1 signaling, we generated NFATc1-knockdown cell lines from BxPC-3 and Mia PaCa-2 pancreatic cancer cells; and overexpressed NFATc1 in Mia PaCa-2 cells. Knockdown or overexpression of NFATc1 gene and protein expression was confirmed by quantitative real-time PCR and western blotting (Fig. 3A).

**Figure 4.**
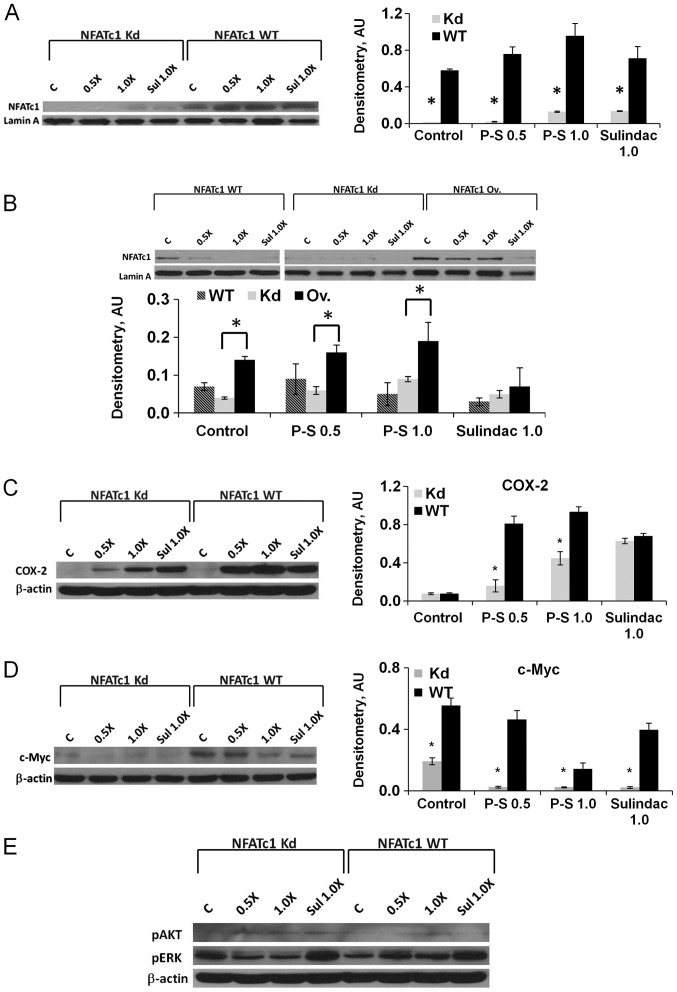
P-S induces nuclear NFATc1 expression and its downstream signaling. (A) BxPC-3 NFATc1 WT and knockdown (Kd) cells were treated with P-S for 24 h, lysed and nuclear NFATc1 was determined by western blotting. (B) Mia PaCa-2 NFATc1 WT, Kd and overexpressing (Ov.) cells were treated with P-S for 24 h, lysed and nuclear NFATc1 was determined by western blotting. (C, D and E) BxPC-3 NFATc1 WT and Kd cells were treated with vehicle, P-S 0.5 and 1.0×IC_50_, or sulindac 1.0×IC_50_ for 24 h. Cells were lysed and the protein expression of COX-2, c-Myc, pAKT and pERK were analyzed by western blotting. ^*^p<0.05, compared to control group.

P-S (0.5×IC_50_ and 1×IC_50_) or sulindac (1×IC_50_) treatment in BxPC-3 and Mia PaCa-2 (NFATc1 WT, knockdown or over-expressing) cells induced the expression of NFATc1 protein (Fig. 3B and C). In addition, nuclear accumulation of NFATc1, indicative of NFATc1 activation, was increased dose-dependently upon treatment with P-S in BxPC-3 and Mia PaCa-2 cells (Fig. 4A and B). In all cases, the protein levels of NFATc1 were lower in NFATc1-knockdown cells compared to wild-type cells, suggesting that shRNA effectively abrogates the induction of NFATc1 by P-S (p<0.05). In summary, P-S induced the expression of NFATc1 in pancreatic cancer cells *in vitro*.

Next, we investigated the expression of c-myc and COX-2, target genes of NFATc1, in pancreatic cancer cells in response to P-S treatment. While no difference in COX-2 protein expression (Fig. 4C) was observed in untreated wild-type and NFATc1-knockdown BxPC-3 cells, P-S treatment induced COX-2 expression in both cell lines, with a more dramatic effect in the wild-type cells. A similar effect was also observed after treatment with sulindac. The expression of c-Myc was suppressed in NFATc1-knockdown cells compared to wild-type (Fig. 4D). In contrast to COX-2, however, c-Myc expression decreased in response to P-S in a concentration-dependent manner; and the effect was greater in NFATc1-knockdown cells. While it is apparent that NFATc1 positively regulates c-Myc expression, the c-Myc suppressing effects of P-S appears to be NFAT-independent.

### NFATc1 expression modulates the anticancer effect of P-S in vitro

Given the importance of NFATc1 in pancreatic carcinogenesis and the effect of P-S in its expression, we examined its role as a modulator of the therapeutic efficacy of P-S in pancreatic cancer. NFATc1-knockdown BxPC-3 and Mia PaCa-2 cells were both sensitized to the cytotoxic effects of P-S, as indicated by the 3- and 2.4-fold reduction in 24 h IC_50_ values compared to the control shRNA-expressing cells (Table II). On the other hand, the cytotoxic effect of P-S is reduced in NFATc1 overexpressing Mia PaCa-2 cells (Table II). These results suggest that NFATc1 expression levels negatively regulate cellular response to P-S. We additionally examined the effect of non-NSAID anticancer agents on BxPC-3 WT and NFATc1-knockdown cells, including: fluorouracil (a pyrimidine analogue), valproic acid (histone deacetylase inhibitor), and irinotecan (topoisomerase poison). Table III shows that for the three drugs tested, an enhanced potency was observed in NFATc1-knockdown cells in comparison to WT (1.6- to 2.2-fold). These findings indicate that NFATc1 protein expression in pancreatic cancer cells decreases the potency of diverse anticancer agents, suggesting that NFATc1 plays a key role in modulating drug response in pancreatic cancer.

### NFATc1 expression modulates the anticancer effect of P-S in vivo

**Figure 5.**
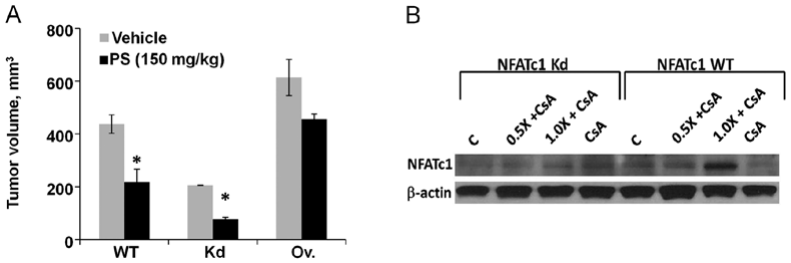
NFATc1 modulates the anticancer effect of P-S and chemotherapeutic drugs. (A) Efficacy of P-S in mice bearing Mia PaCa-2 wild-type (WT), NFATc1-knockdown (Kd), or NFATc1 overexpressing xenografts. Mice were treated with vehicle or P-S 100 mg/kg/day. (B) BxPC-3 NFATc1 WT and Kd cells were treated with vehicle, P-S 0.5×IC_50_ + CsA and 1.0×IC_50_ + CsA, or CsA alone for 24 h. NFATc1 protein expression was analyzed by western blotting. ^*^p<0.05, compared to control group.

We determined the effect of NFATc1 knockdown or overexpression on the tumor response to P-S *in vivo*. Following a prevention protocol, animals were first treated with P-S for one week prior to subcutaneous injection of wild-type, NFATc1-knockdown and NFATc1-overexpressing Mia PaCa-2 cells. Animals were then treated for 4 more weeks. As shown in Fig. 5A, tumor volume was significantly reduced by 50.4% in P-S-treated wild-type compared to the vehicle (p=0.013). In NFATc1 knockdown tumors, P-S treatment caused a 62.8% reduction in tumor volume compared to the vehicle (p=0.0005). In contrast, P-S alone had a weak inhibitory effect (25.9% inhibition) on tumors overexpressing NFATc1 and the effect was not statistically significant. Consistent with *in vitro* findings, NFATc1 expression is an important factor that negatively regulates the tumor responsiveness to P-S *in vivo*.

### Pharmacological targeting of NFATc1 sensitizes pancreatic cancer cells to the cytotoxic effect of P-S

The immunosuppressive drug cyclosporin A (CsA) is an inhibitor of NFAT-mediated transcription activity. CsA prevents dephosphorylation of NFAT by calcineurin, leading to sequestration of phosphorylated NFAT in the cytoplasm. To analyze the effect of NFATc1 inhibition on cellular response to P-S, BxPC-3 wild-type and NFATc1-knockdown cells were pre-treated with CsA (1 *μ*M for 1 h), followed by either 0.5× or 1.0×IC_50_ P-S for 24 h. A slight (1.2-fold) enhancement of potency of P-S was observed in combination with CsA in wild-type cells, while no effect was observed in NFATc1-knockdown cells (data not shown). Western blot analysis showed a significant reduction in the protein levels of NFATc1 in both the wild-type and NFATc1 knockdown cells treated with CsA alone or P-S plus CsA (Fig. 5B). P-S increased the expression of NFATc1, while CsA reduced it. These results suggest that CsA antagonizes the induction of NFATc1 by P-S, thereby potentiating the cytotoxic effect of P-S.

## Discussion

Pancreatic cancer is among the most lethal of human cancers and it is highly resistant to many chemotherapeutic drugs. Our findings demonstrate that P-S possesses considerable efficacy in the pre-clinical models of pancreatic cancer while being apparently safe, establish that NFATc1 is a critical factor in mediating drug resistance in pancreatic cancer, and that targeting NFATc1 improves tumor response to chemotherapeutic drugs, including P-S.

In a panel of four human pancreatic cancer cell lines (*Kras* wild-type or mutant), P-S was consistently more potent (19- to >100-fold) than sulindac in inhibiting their growth. The antitumor efficacy of P-S was also established in a pancreatic cancer xenograft model which encompass *Kras* wild-type (BxPC-3) and mutant (Mia PaCa-2) human pancreatic cancer cell lines. In both cases, P-S significantly inhibited the growth of the xenografts compared to control, irrespective of the *Kras* mutation status. Apart from efficacy, P-S also exhibits a favorable safety profile, evidenced by the apparent lack of organ toxicity, and more importantly, minimal gastrointestinal side effects, a dose-limiting toxicity of its parent NSAID sulindac (10).

Underlying the growth inhibitory potency of PS was its combined cytokinetic effect consisting of suppressed proliferation and enhanced apoptosis. Such an effect was observed both *in vitro* and *in vivo*. Induction of oxidative stress is a key mechanism of action for several anticancer agents, including P-S. As cancer cells have elevated ROS generation and are under increased intrinsic oxidative stress, these cells are more vulnerable to further oxidative insults induced by ROS-generating agents. Consistent with previous reports (10,16,18,19,22), there is a significant increase in ROS in response to P-S in pancreatic cancer cells. The cytokinetic effect and induction of ROS contributes to the antitumor effect of P-S in pancreatic cancer.

Our study also unravels a novel role of NFATc1 in mediating drug resistance in pancreatic cancer. P-S triggered profound upregulation of NFATc1 and its nuclear translocation in pancreatic cancer cells, leading to robust induction of its transcriptional targets, including COX-2. It is conceivable that such an induction of pro-proliferative and pro-survival factors by NFATc1 has important implications for drug response and resistance. Indeed, genetic silencing of NFATc1 in pancreatic cancer cells enhanced the potency of P-S; while its ectopic expression conferred drug resistance. Accordingly, NFATc1-knockdown Mia PaCa-2 xenografts were notably more responsive to P-S compared to wild-type, whereas NFATc1-overexpressing xenografts were insensitive to P-S. This observation was extended to mechanistically diverse anti-cancer agent (5-FU, valproic acid and irinotecan); suggesting that NFATc1 promotes a broad resistance to chemotherapeutic drugs in pancreatic cancer.

Given the important role of NFATc1 in drug resistance, it represents a novel prognostic factor for predicting drug response and a potential therapeutic target for improving tumor responsiveness to chemo-therapeutic drugs. NFATc1 activation is regulated by Ca^2+^/calcineurin signaling pathway (23). Fluctuation in Ca^2+^ levels simulates calcineurin to dephosphorylate NFATc1, which then translocates to the nucleus to activate gene expression. Pharmacological inhibition of calcineurin by CsA blocks NFATc1 activation as well as its nuclear translocation; and more importantly, CsA treatment sensitizes pancreatic cancer cells to P-S. Our findings thus provide a biochemical basis for synergism between P-S and CsA in pancreatic cancer, and suggest that the combination therapy with P-S and NFATc1 inhibitors may be a promising chemotherapeutic approach to improve treatment outcomes.

**Figure 6.**
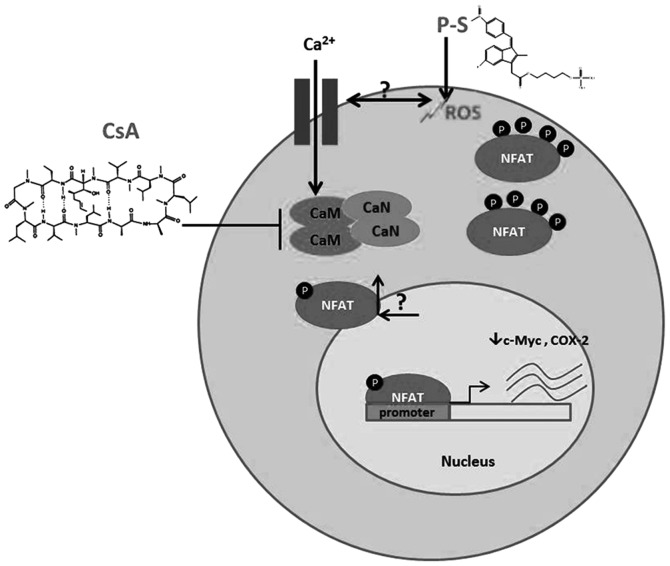
Proposed mechanistic interaction of P-S and NFATc1. PS induces ROS, followed by the dephosphorylation of NFATc1, which in turn, leads to increased cytoplasmic and nuclear NFATc1 protein expression. Activation of NFATc1-mediated transcription increased the expression of c-Myc and COX-2. When PS is combined with CsA, the latter reduces the induction of nuclear and cytoplasmic NFATc1 protein expression. CsA can be conceptualized in this diagram as affecting calcium homeostasis, which in turn modulates the activity of NFATc1. Thus, PS and CsA may synergize to inhibit pancreatic carcinogenesis.

In conclusion, our study demonstrates that P-S is a promising agent that can effectively inhibit *Kras* wild-type and *Kras*-mutant pancreatic cancer *in vitro* and *in vivo*. We uncovered a novel role of NFATc1 in modulating drug response in pancreatic cancer, and proposed a pharmacological approach to overcome the drug resistance associated with NFATc1 activation (outlined in Fig. 6). Overall, the effectiveness and safety of P-S in the treatment of pancreatic cancer suggest that this compound merits further evaluation.

## Figures and Tables

**Table I.** The 24-h IC_50_ values of P-S and sulindac in a panel of pancreatic cancer cell lines.

Cell line	P-S IC_50_, *μ*M/potency enhancement^a^		

	24 h	48 h	72 h
BxPC-3	106±4 >19	42±2 >48	15±2 >133
Mia-PaCa-2	79±2 >25	60±2 >33	55±1 >36
Panc-1	99±2 >20	83±4 >32	51±2 >39
HPAF-II	76±2 >26	65±2 >31	57±1 >35

a Over sulindac whose IC_50_ was consistently >1,000 *μ*M for all cell lines.

**Table II.** Effect of NFATc1 knockdown or overexpression on 24-h IC_50_ values of P-S in BxPC-3 and MIA PaCa-2 cells.

Cell line	P-S IC_50_, *μ*M		

	Mock transfected	NFATc1 knockdown	NFATc1 overexpression
BxPC-3	104±2	35±2	ND
Mia-PaCa-2	93±1	38±2	111±2

ND, not determined.

**Table III.** Effect of NFATc1 knockdown on 24-h IC_50_ values of chemotherapeutic drugs in BxPC-3 cells.

Drug	P-S IC_50_, *μ*M		

	Mock transfected	NFATc1 knockdown	Potency enhancement
Fluorouracil	586±2	263±2	2.2
Valproic acid	543±1	338±3	1.6
Irinotecan	58.5±4	26.8±1	2.2
